# Healthcare-associated COVID-19: The experience of an academic medical center

**DOI:** 10.1017/ice.2020.1357

**Published:** 2020-12-07

**Authors:** Rachel J. Pryor, Michelle Doll, Michael P. Stevens, Kaila Cooper, Emily J. Godbout, Oliva Hess, Gonzalo Bearman

**Affiliations:** 1 Hospital Infection Prevention Program, Virginia Commonwealth University, Richmond, Virginia; 2 Division of Infectious Diseases, Virginia Commonwealth University, Richmond, Virginia; 3 Division of Pediatric Infectious Diseases, Children’s Hospital of Richmond at Virginia Commonwealth University, Richmond, Virginia

*To the Editor*—Due to fear of transmission of severe acute respiratory coronavirus virus 2 (SARS-CoV-2) in the healthcare setting, individuals may delay seeking healthcare until symptoms are too severe to ignore, possibly leading to worse clinical outcomes.^1,2^ We assessed healthcare-associated (HA) SARS-CoV-2 transmission in an academic medical center within a region of moderate community coronavirus disease 2019 (COVID-19)^3^ to determine whether our infection prevention practices are effective in preventing HA SARS-CoV-2 transmission.

Virginia Commonwealth University Health System (VCUHS) is an 865-bed institution in Richmond, Virginia. We tested our first COVID-19 person under investigation (PUI) on March 2, 2020. The first patient diagnosed with COVID-19 was admitted on March 13, 2020. We began universal SARS-CoV-2 screening by polymerase chain reaction (PCR) testing on April 27, 2020. Known SARS-CoV-2–positive patients are isolated in single-occupancy isolation rooms or airborne isolation rooms (if receiving aerosolizing therapies or procedures). Our institution did not implement universal particulate filter respirator (PFRN95) masking for all healthcare workers. PFRN95 masks are only used when there is a concern for aerosolization. Droplet masks and face shields are universally required for all other patient care.

The VCUHS Hospital Infection Prevention Program (HIPP) utilized our institution’s COVID-19 patient database to extract SARS-CoV-2 testing data. We reviewed data of patients with initial negative admission SARS-CoV-2 PCR screen followed by a repeated, positive screen >24 hours into hospitalization. We defined a probable HA COVID-19 case using previously published criteria.^4^ Healthcare-associated COVID-19 could be a (1) case in which symptoms began on hospital days 3–7 in a patient with a known COVID-19 exposure on hospital days 1 or 2 and no known COVID risks prior to hospitalization or (2) a case in which symptoms and the first positive COVID-19 test occurred on hospital day 8–14. We defined a confirmed case of HA COVID-19 as a case in which the first positive COVID-19 test occurred after day 14 of hospitalization.^5^

We reviewed the medical records of each potential HA COVID-19 patient. Patients were excluded if they were known to have been SARS-CoV-2 positive in the past although their first tests on admission were negative (believed to be false-negative results). Patients were also excluded when they were tested for placement prior to discharge, resulting in a positive test but subsequently had 2 negative tests, each 24 hours apart. These positive tests were likely false-positive results. The VCU Institutional Review Board qualified this study for exemption.

From March 2, 2020, through September 30, 2020, 18,814 patients were admitted to our facility. Of these inpatients, 11,482 received SARS-CoV-2 tests, and 723 patients were diagnosed with COVID-19. We identified 21 patients with an initial negative SARS-CoV-2 test that later converted to a positive. Moreover, 9 patients were excluded from our final analysis: 5 patients were excluded because they were known to have had positive tests in the past and 4 patients were excluded because of an initial positive SARS-CoV-2 discharge placement test followed by 2 serial negative tests within 48 hours. Overall, 12 patients were included in our analysis and were classified as probable or confirmed diagnoses of HA COVID-19 (12 of 11,482, 0.10%) (Table 1).

**Table 1.** Summary of Probable and Confirmed Healthcare-Associated (HA) COVID-19 in an Academic Medical Center

Days Between Admission and First Positive Test	HA vs CA	Age	Gender	Reason for Repeated SARS-CoV-2 Testing	Case Information
5	Probable CA	77	Female	Screening for discharge placement	Asymptomatic. Admitted after fall in SNF. Repeated SARS-CoV-2 screen ordered for discharge placement. Repeated test was positive. Hospital epidemiology recommended additional testing. Third test was negative, but the fourth test was again positive.
6	Probable CA	72	Female	Respiratory decompensation	Admitted for acute hypoxic respiratory failure. Due to negative test on admission, hypoxia was believed secondary to a cardiac condition. Patient decompensated on day 6 of hospitalization. A repeated SARS-CoV-2 test was positive.
6	Probable CA	55	Male	Ongoing fevers	Presented with subacute febrile illness with dyspnea and flank pain. Initial SARS-CoV-2 test was negative. Remained febrile throughout hospitalization; SARS-CoV-2 testing was performed on bronchoscopy specimen and was positive despite prior negative tests performed from nasopharyngeal specimens collected in our facility and twice at an OSH.
7	Probable CA	89	Male	Discharge placement	Admitted from LTCF for altered mental status; SARS-CoV-2 test was negative. Tested again for placement; SARS-CoV-2 test was positive.
4	Probable HA	40	Female	Potential exposure to COVID+ during hospitalization	Admitted for perinephric hematoma and was believed to have been exposed to a SARS-CoV-2–positive patient. Retested and was SARS-CoV-2 positive.
8	Probable HA	78	Female	Respiratory decompensation	Admitted for mesenteric ischemia; SARS-CoV-2 test was negative. Rapidly decompensated on day 8 of hospitalization; retested and was positive.
8	Probable HA	65	Male	Screening for discharge placement	Transferred from OSH for evaluation of subdural hematoma. Retested for placement and was positive.
9	Probable HA	39	Female	Screening for discharge placement	Admitted for dyspnea and hypoxia believed to be due to myotonic dystrophy since initial SARS-CoV-2 test was negative. Retested for placement and was positive.
10	Probable HA	31	Male	Fever of unknown origin, exposed to COVID+ during hospitalization	Presented to OSH 3 days after being in a motor vehicle accident. Transferred to our facility. Alcohol dependent, was intubated for withdrawal. Persistent fevers coupled with likely hospital exposure led to a repeated test for SARS-CoV-2; the test was positive.
10	Probable HA	32	Male	Screened prior to surgery	Admitted following motorcycle crash and was negative for SARS-CoV-2. Retested prior to surgery and was positive.
22 (16 days at OSH and 6 days at our facility)	Confirmed HA	68	Male	Respiratory decline with fevers; known positive contacts, but with two negative COVID tests	Presented to OSH with SOB, dyspnea and fever but with negative SARS-CoV-2 test (despite known SARS-CoV-2–positive contacts). Had a complicated stay at OSH. Eventually transferred to our facility for a higher level of care and concerns for a hepatic abscess. First SARS-CoV-2 test at our facility was also negative. However, test on day 7 was positive.
25	Confirmed HA	69	Female	Respiratory decompensation	Admitted with cholecystitis, complicated hospital stay. Retested after respiratory status worsened and was positive.

Note. CA, community associated; SNF, skilled nursing facility; OSH, outside hospital; LTCF, long-term care facility; SOB, shortness of breath.

Although the exact community prevalence of COVID-19 is unknown, a recent study by the Virginia Department of Health reported that 2.4% of Virginia adults have SARS-CoV-2 antibodies,^6^ suggesting an overall low prevalence of COVID-19 in Virginia. Over the study period, 723 number of patients were admitted to our institution with COVID-19. In contrast to a recent publication from a healthcare system in the northeastern United States with a different study period, the COVID-19 admission prevalence was lower at our institution: 723 of 11,482 (6.3%) versus 697 of 7,394 (9.4%).^5^

The strengths of this study include an in-depth medical record review of all patients who converted from a negative to positive SARS-CoV-2 test result during their hospital stay. Furthermore, all PCR testing for SARS-CoV-2 was performed within our institution, allowing for consistency in process. We also employed previously published definitions for probable and confirmed HA COVID-19.

This study has several limitations. We excluded patients who may have been diagnosed with COVID-19 after their hospitalization; postdischarge surveillance is not routinely performed. Only patients who require SARS-CoV-2 testing for discharge placement are tested prior to discharge, so unless a patient develops symptoms, has a known exposure, or needs placement, they are not retested. Thus, cases of HA COVID-19 may have been undetected. We cannot know with certainty whether the patients determined to have false-positive tests were not reinfected with SARS-CoV-2, which led to their test conversion while hospitalized. Based on our limited knowledge of asymptomatic carriers of SARS-CoV-2, we cannot know with certainty whether patients who we excluded for false-positive results truly did not have COVID-19 with intermittently detectable viral loads. Finally, because not every patient admitted to our hospital underwent SARS-CoV-2 testing, we were unable to determine the exact prevalence among our patient population.

Our institution is in a region of lower COVID-19 prevalence, and PFRN95 masks are not used for universal patient care. Our infection prevention strategy included admission screening for SARS-CoV-2 and patient isolation with droplet or airborne precautions and contact precautions for COVID-19 suspected or confirmed cases. Face shields were universally required with droplet or PFRN95 masks, and door monitors were employed to ensure consistency of PPE donning and doffing. These processes and outcomes provide growing assurance that infection prevention protocols are adequate to prevent the transmission of SARS-CoV-2 in the acute-care setting, allowing for the safe provision of necessary patient care despite an ongoing pandemic.
